# Classics in Chemical Neuroscience: Medetomidine

**DOI:** 10.1021/acschemneuro.4c00583

**Published:** 2024-10-15

**Authors:** Pedro de Andrade Horn, Tomayo I. Berida, Lauren C. Parr, Jacob L. Bouchard, Navoda Jayakodiarachchi, Daniel C. Schultz, Craig W. Lindsley, Morgan L. Crowley

**Affiliations:** † Warren Center for Neuroscience Drug Discovery and Department of Pharmacology, Vanderbilt University, Nashville, Tennessee 37232, United States; ‡ Department of Chemistry, and Department of Biochemistry, Vanderbilt University, Nashville, Tennessee 37232, United States

**Keywords:** Medetomidine, Dexmedetomidine, α_2_-Adrenoreceptors, Fentanyl, Opioid, Veterinary Medicine

## Abstract

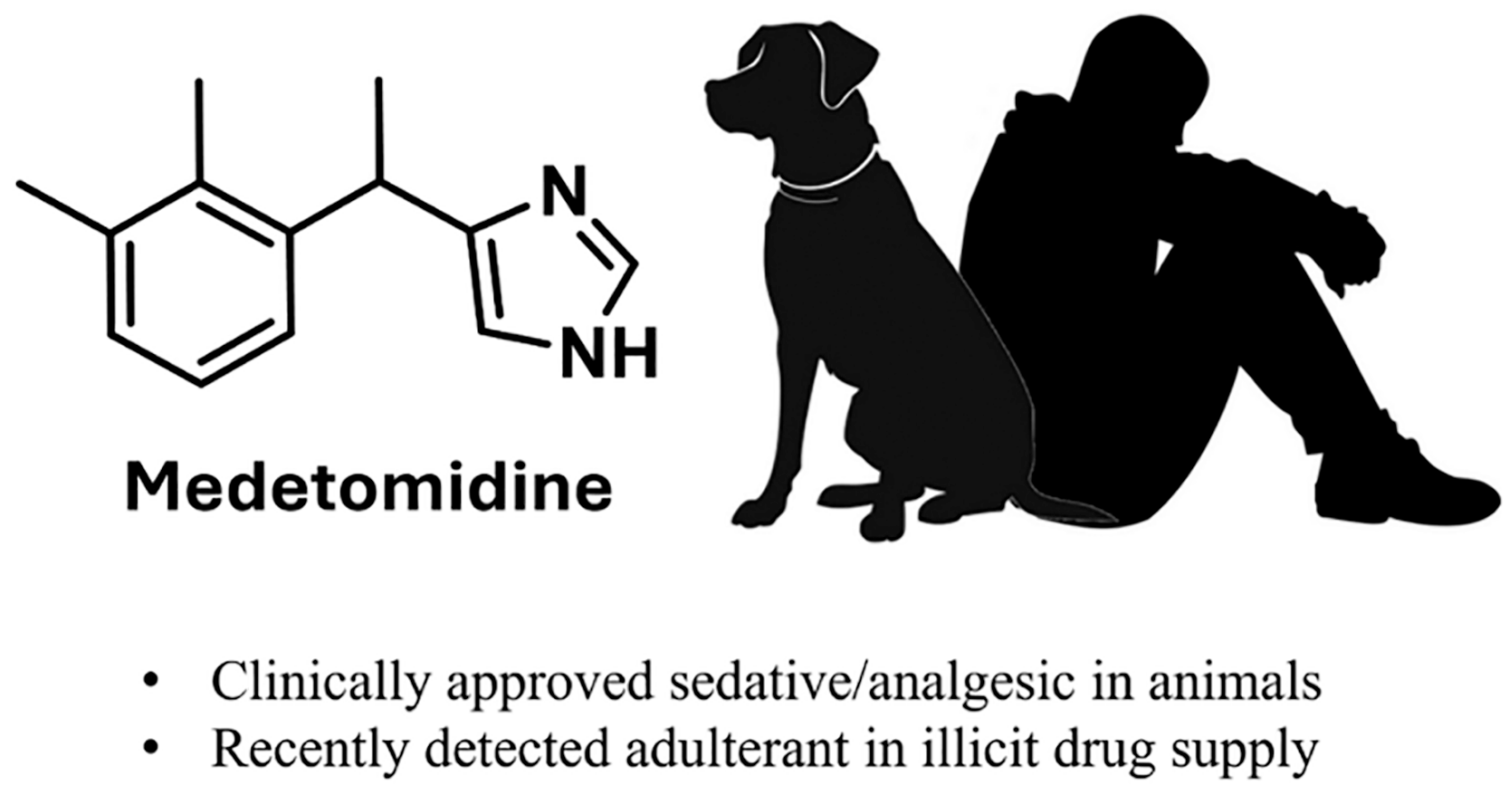

Medetomidine is an FDA-approved α_2_-adrenoreceptor (α_2_-AR) agonist used as a veterinary sedative due to its analgesic, sedative, and anxiolytic properties. While it is marketed for veterinary use as a racemic mixture under the brand name Domitor, the pharmacologically active enantiomer, dexmedetomidine, is approved for sedation and analgesia in the hospital setting. Medetomidine has recently been detected in the illicit drug supply alongside fentanyl, xylazine, cocaine, and heroin, producing pronounced sedative effects that are not reversed by naloxone. The pharmacological effects along with the low cost of supply and lack of regulation for medetomidine has made it a target for misuse. Since 2022, medetomidine has been found as an adulterant in samples of seized drugs, as well as in toxicological analyses of patients admitted to the emergency department after suspected overdoses across several U.S. states and Canada. This Review will discuss the history, chemistry, structure–activity relationships, drug metabolism and pharmacokinetics (DMPK), pharmacology, and emergence of medetomidine as an adulterant in drug mixtures in the context of the current opioid drug crisis.

## Introduction

**Figure 1:**
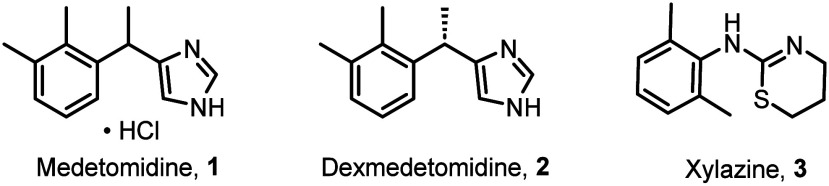
Chemical structures of selected α_2_-AR agonists.

The opioid crisis has evolved over the last few decades, starting with the rise in prescription opioid overdose deaths, followed by an increase in heroin associated fatalities.^1^ In the past decade, the shift from heroin to fentanyl has resulted in a dramatic surge in opioid-related deaths and cases of opioid use disorder.^2^ This alarming trend prompted the Department of Health and Human Services to declare a public health emergency in 2017.^2,3^ The severity of the crisis has continued to escalate, with opioid-related overdose deaths rising dramatically from 49,860 in 2019 to 81,806 in 2022, marking a 64% increase.^4^ As the crisis evolves, new issues have emerged in the illicit drug supply. One concerning development is the rise in the number of different adulterants being found in street drugs, which has shifted the crisis from small, local outbreaks to larger regional ones. Recently, medetomidine, a potent α_2_-adrenergic receptor (α_2_-AR) agonist typically used for veterinary sedation and anesthesia, has been identified as one of the latest adulterants appearing in illicit drug markets, adding another layer of risk to an already deadly situation (Figure 1).^5−7^

Medetomidine, ((±)-4-(1-(2,3-dimethylphenyl)ethyl)-1*H*-imidazole), was developed by Farmos Group Ltd. and first brought to market in Europe as Domitor in 1987.^8^ In 1993, Farmos Group Ltd. merged with Orion Pharmaceuticals, who continued development of this drug, leading to its first FDA approval in 1996 for use in dogs over 12 weeks old.^9^ Since its initial approval, it has found application in other animals such as cats and sheep.^10−12^ Medetomidine is marketed as an HCl salt and a racemic mixture of the dexmedetomidine (also termed dextro-medetomidine) and levo-medetomidine stereoisomers, with the dextro-isomer being the active component at pharmacologically relevant doses.^10,13^ Dexmedetomidine (marketed as Dexdor or Precedex), is twice as active as medetomidine.^14^ It was approved by the FDA for human use in 1999 for sedation of intensive care unit (ICU) patients who are intubated and mechanically ventilated.^15^ Since the initial approval, it has garnered additional indications including use as an analgesic and in nonintubated patients before and during surgical and other procedures.^16^ In addition to its veterinary use, medetomidine has also found use as an antifouling agent in marine paints.^17−19^

As an α_2_-AR agonist, medetomidine centrally induces sedation by decreasing the noradrenergic activity in the locus coeruleus^14,20^ and acts both centrally and peripherally to induce its analgesic effect.^20,21^ While the pharmacological properties of medetomidine have secured its approval as an indispensable veterinary anesthetic, they have also rendered it an attractive adulterant. Recent reports have identified medetomidine in street drug mixtures, often combined with opioids like fentanyl, other α_2_-AR agonists such as xylazine, and stimulants including cocaine and methamphetamine.^22,23^ The combination of xylazine and fentanyl, commonly referred to as “Tranq” or “zombie drug,″ in addition to the incorporation of medetomidine has emerged as a significant public health concern. The rationale behind these dangerous mixtures likely stems from the ability of α_2_-AR agonists like medetomidine to potentiate the sedative and euphoric effects of opioids. Additionally, the low cost and lack of regulation of these veterinary tranquilizers further exacerbates the ongoing opioid crisis.^23,24^

Medetomidine and xylazine belong to the same drug class, namely α_2_-AR agonists.^20^ However, medetomidine is over 100 times more potent and selective for α_2_-AR than xylazine, and it is associated with more severe hemodynamic instability associated with this drug class, including bradycardia, hypotension, and CNS depression.^25,26^ The presence of medetomidine in illicit drugs poses significant health risks to users, and overdose can be fatal. This is worsened by the fact that medetomidine’s effects cannot be reversed by naloxone, an opioid antagonist administered during suspected opioid overdoses.^27^ Overdoses involving medetomidine are continuing to become more widespread, and clandestine laboratory seizures have occurred in Ohio, Florida, and Canada.^28^ The increasing presence of medetomidine in the illicit drug supply has prompted this review to summarize the literature surrounding the chemistry, pharmacology, and pharmacokinetics of medetomidine.

## Chemistry

**Scheme 1:**
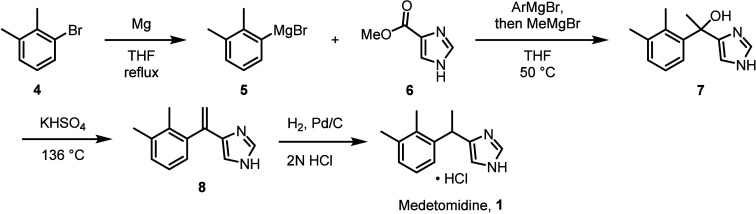
First Synthesis of Medetomidine Hydrochloride

Medetomidine, or (±)-4-(1-(2,3-dimethylphenyl)ethyl)-1*H*-imidazole (CAS: 86347–14–0; C_13_H_16_N_2_), is a 4-substituted imidazole traditionally prepared as its hydrogen chloride salt (white solid, mp 175.5–178.5 °C).^29^ It was first disclosed in a patent filed by the Finnish company Farmos Group Ltd. in 1981 (first published in 1983), which detailed the preparation of various substituted imidazoles and their antihypertensive, antithrombotic, and antifungal properties.^30,31^ Briefly, the inventors synthesized medetomidine hydrochloride through sequential Grignard reactions to afford tertiary alcohol **7**, which was then dehydrated and hydrogenated to give the desired product **1** in 17% overall yield (Scheme 1).^31^

**Scheme 2:**
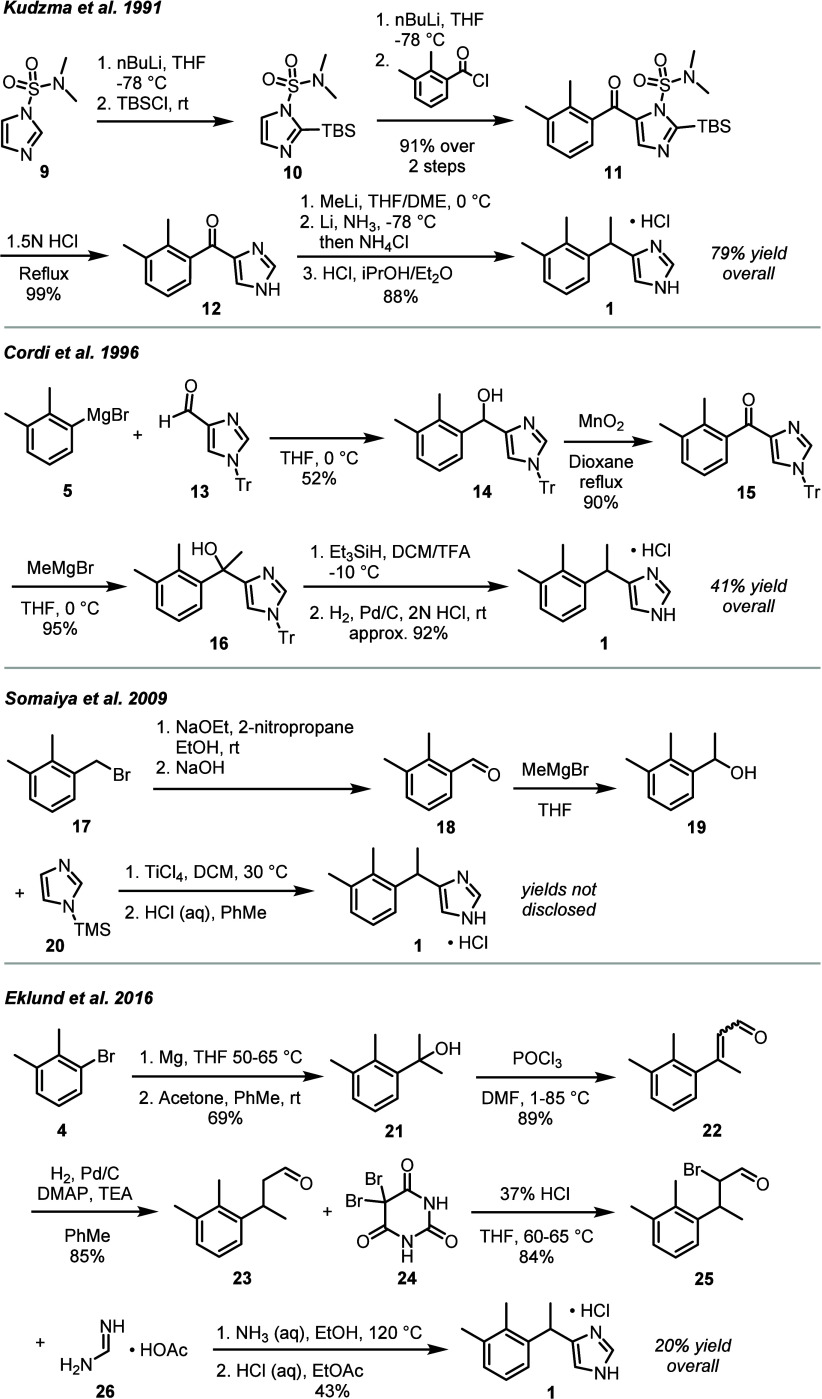
Selected Synthetic Routes for Medetomidine Hydrochloride

Since its initial publication, additional synthetic routes have been developed for the synthesis of medetomidine with the intent to improve overall yield, minimize the use of toxic or dangerous reagents, or avoid transformations with poor scalability, selected examples of which are depicted in Scheme 2.^32−35^

**Scheme 3:**
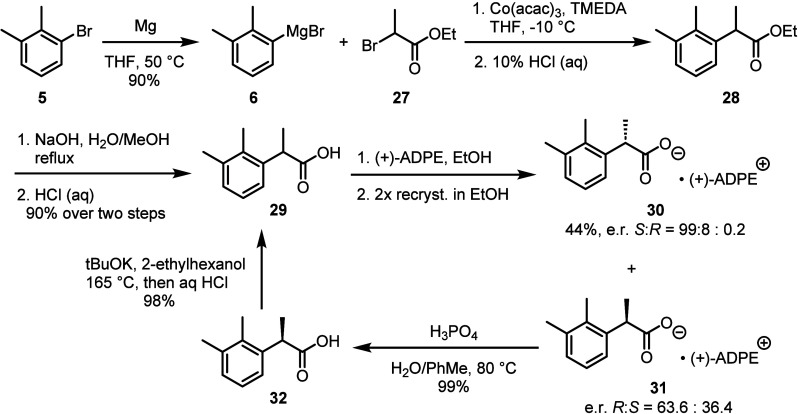
Early-Stage Chiral Resolution of a Key Intermediate for the Synthesis of Dexmedetomidine with the Capability to Recycle the Undesired Enantiomer^48^, (+)-ADPE = (1*S*,2*R*)-(+)-2-amino-1,2-diphenylethanol.

As the active isomer of this pharmaceutical agent is the *S*-enantiomer, significant efforts have been made toward the chiral resolution or stereoselective synthesis of dexmedetomidine.^33,36−48^ Farmos Group Ltd., who published the first synthesis of medetomidine, also published the first procedure for the chiral resolution of dexmedetomidine, which required multiple recrystallizations of racemic medetomidine with (+)-tartaric acid to afford the enantiopure product.^36,37^ Methods for the synthesis of dexmedetomidine involve catalytic enantioselective hydrogenation of alkene **8** or its protected derivative using various chiral phosphine ligands.^43−47^ These methods face significant limitations, however, as the undesired enantiomer (levo-medetomidine) is discarded during the course of chiral resolution, and enantioselective hydrogenations require expensive catalysts to achieve sufficient enantiomeric excess (*ee*).^43−47,49^ To mitigate this, VIC Animal Health recently disclosed a process chemistry route wherein intermediate **30** could be selectively crystallized in high *ee* using a chiral amine, and the undesired isomer **31** could be readily recycled and reintroduced into the synthetic route (Scheme 3).^48^

## SAR

While a large number of analogs of medetomidine were published in its initial patent,^31^ to our knowledge, no biological data for these structures have been made publicly available. However, it can be reasonably assumed that the modifications outlined in the patent result in less desirable compounds (less potent, less selective, etc.) since they were not carried forward as the final drug candidate. Beyond this initial patent, several academic groups have published limited structure–activity relationship (SAR) on medetomidine (highlighted below).

**Figure 2:**
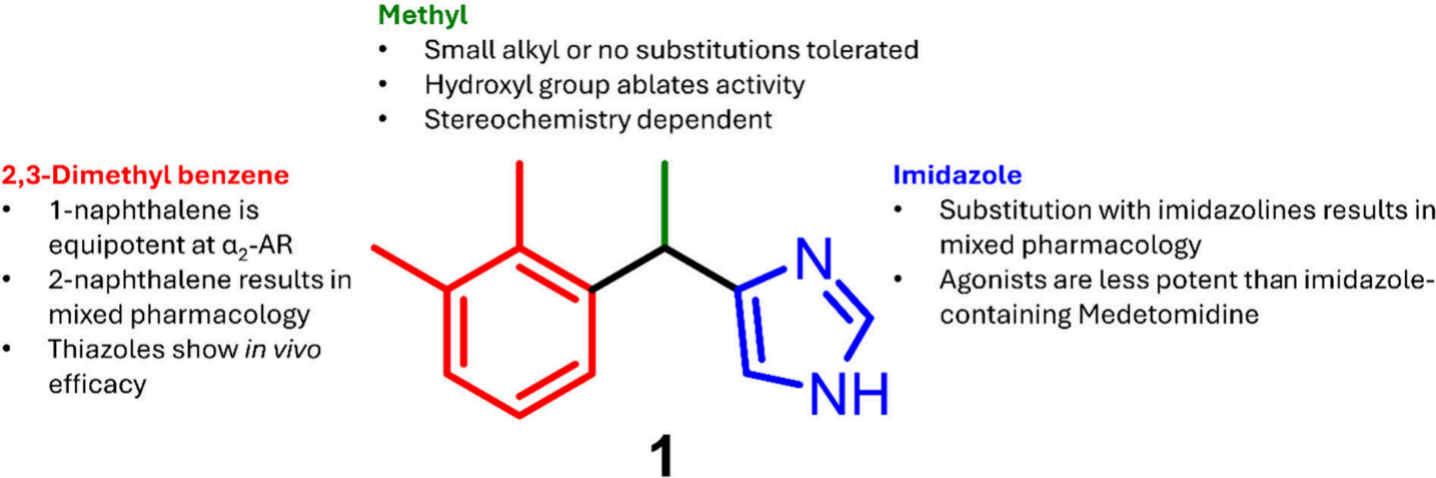
SAR summary of medetomidine.

The structure of medetomidine consists of an imidazole linked to a 2,3-dimethyl benzene moiety through a methylene bridge containing a methyl group at the α position (Figure 2). Other smaller alkyl substituents at the α position are fairly well tolerated, however none offered superior potency compared to the methyl substituent. Conversely, removal of this methyl group or hydroxyl substitutions at this position led to decreased agonist potency.^50^ As previously mentioned, the active isomer of medetomidine is its *S*-enantiomer, and this stereochemical trend holds true for other α_2_-AR agonists.^51^ This suggests that interactions between ligands and α_2_-ARs are both conformationally and structurally dependent. Molecular docking studies and structural analysis of reported α_2_-AR ligands suggest there is limited opportunity to expand the chemical space for agonists at α_2_-AR due to its constrained binding pocket.^52^

Replacement of the 2,3-dimethylphenyl group with a 1-naphthalene moiety resulted in equipotent activity in a human platelet aggregation assay, while improvement in activity was observed when employing a 2-naphthalene group. Generally, the *R*-enantiomers of the 2-naphthalene analogs were less potent α_2_-AR agonists than their *S* counterparts.^53^ Additionally, α_2_/α_1_ selectivity was increased in *des*-methyl versions of 1-naphthalene-substituted compounds.^53^ Replacement of the benzene moiety with substituted thiazoles was also tolerated, resulting in compounds that exhibited low nanomolar binding affinity to α_2_-AR (K_i_ = 8–28 nM) and were efficacious in murine pain models.^54^ Replacement of the imidazole with imidazolines resulted in an array of both α_1_-AR and α_2_-AR activities, but none of the analogues surpassed medetomidine’s α_2_-AR agonist potency.^55^

More recently, Fink et al. published novel α_2_-AR ligands with low nanomolar affinity from a large virtual screening program of over 300 million molecules, however these compounds lack α_2_-AR subtype selectivity.^56^ Chromane derivatives have also recently been reported with low nanomolar affinity and subtype selectivity for α_2_-AR compared to dexmedetomidine.^52^ In addition to SAR campaigns to influence α_2_-AR effects on potency and selectivity, modifications have been explored to increase its utility as a tool compound. Introduction of a photolabile CF_3_-diazirine group to the benzene ring resulted in a photoaffinity label for the α_2_-AR that still retained potency at the receptor.^57^

## Drug Metabolism and Pharmacokinetics

Medetomidine and dexmedetomidine have been extensively studied in human and veterinary medicine. Dexmedetomidine is approved in humans for intravenous (IV) infusion and has also been studied in the context of intramuscular, intranasal, buccal, and oral administration. While dexmedetomidine is generally well-tolerated through extravascular administration, its oral bioavailability is only 16%, likely due to extensive first-pass metabolism in the liver.^58^ In healthy volunteers, dexmedetomidine demonstrates linear kinetics when dosed at 0.2–0.7 μg/kg/h via IV infusion for up to 24 h.^15^ Following infusion, dexmedetomidine is rapidly distributed, with a distribution half-life of 2.5–6 min and a steady-state volume of distribution ranging from 72–194 L.^58−61^ It is highly protein-bound in both men and women, with an average binding rate of 93.7%.^62^ Dexmedetomidine is cleared at a rate of 30 to 53 L/h, with a terminal half-life of 2 to 2.5 h.^58−61^

Dexmedetomidine is extensively metabolized in the liver, with a hepatic extraction ratio of 0.7.^60^ It is primarily metabolized through cytochrome P450s (CYPs), specifically CYP2A6, and UDP-glucuronosyltransferases (UGTs), including UGT1A4 and UGT2B10, resulting in direct hydroxylation and glucuronidation, respectively.^62,63^ After an IV infusion of 2 μg/kg [^3^H]dexmedetomidine in humans, it was determined that the major circulating metabolites are *N*-glucuronides and *O*-glucuronides, which account for 41% and 24% of the plasma AUC radioactivity, respectively, while the unchanged parent compound constitutes 15% of plasma radioactivity.^62^ Following biotransformation, dexmedetomidine is primarily eliminated by the kidneys, with 95% of metabolites excreted in the urine and 4% in the feces.^62^

With respect to medetomidine’s use in veterinary medicine, a single subcutaneous dose study of 80 μg/kg of [^3^H]medetomidine in rats, dogs, and cats demonstrated rapid distribution and high brain penetration, with peak concentrations observed within 30 min of administration.^64^ Dogs and cats exhibited similar apparent volumes of distribution (2.8 and 3.5 L/kg, respectively), clearance rates (27.5 and 33.4 mL/min/kg, respectively), and elimination half-lives (0.97 and 1.6 h, respectively).^64^ Rats exhibited higher volume of distribution (8.2 L/kg) and clearance rates (88.5 mL/min/kg) compared to dogs and cats, with similar elimination half-lives (1.09 h).^65^ In dogs, medetomidine and dexmedetomidine have similar clearance rates, volume of distribution at steady-state, and terminal half-life when administered as an IV bolus of either 40 μg/kg or 20 μg/kg.^66^ In preclinical species, medetomidine is primarily metabolized through hydroxylation by CYPs.^66,67^ Elimination is primarily through the kidneys and only rats showed significant excretion through the feces.^64^

**Table 1:** Known Pharmacokinetic Parameters of Medetomidine in Selected Species^64,65^

species	volume of distribution (L/kg)	clearance (mL/min/kg)	*t*_1/2_ (hr)
dog	2.8	27.5	0.97
cat	3.6	33.4	1.6
rat	8.2	88.5	1.09

## Molecular Pharmacology

Medetomidine is a selective and potent α_2_-AR agonist. α_2_-ARs are G_i/o_ G-protein coupled receptors that exist as three isoforms: α_2A_, α_2B_, and α_2C_. These receptor subtypes display high sequence homology and differ in their distribution and density throughout the central and peripheral nervous system.^68^ α_2A_- and α_2C_-ARs are primarily located throughout the CNS and are responsible for producing sedation, analgesia, and sympatholytic effects, while α_2B_-ARs are predominantly found on vascular smooth muscle and produce vasopressor effects.^20,69^ This difference in α_2_-AR location, distribution, density, and species difference has led to variability in the drug dose and effects seen with medetomidine administration.^10,70^ The endogenous ligands for the α_2_-ARs are catecholamines, specifically norepinephrine and epinephrine. Agonist binding initiates a downstream signaling cascade that results in the inhibition of various pathways that result in a decrease in intracellular cAMP and calcium levels. Physiologically, agonist activation results in inhibition of norepinephrine release via hyperpolarization of norepinephrine-producing neurons in a negative feedback manner, resulting in sedation, analgesia, muscle relaxation, and anxiolysis.^71^ In response to the decrease in sympathetic tone, undesirable side effects are observed including bradycardia, bradyarrhythmia, bradypnea, hypothermia, and decreased cardiac output.^10,72^

To date, medetomidine is the most selective α_2_-AR agonist used both in veterinary medicine and clinically (as dexmedetomidine), with a selectivity ratio (α_2_/α_1_) of 1620 (Table 2).^26,73^ This is in contrast to the more recent α_2_-AR adulterant found in the illicit drug supply, xylazine (α_2_/α_1_ = 160), and the well-known antihypertensive agent clonidine (α_2_/α_1_ = 220). Stimulation of α_1_-ARs has been shown to lead to arousal, increased locomotor activity, and cardiovascular effects in rats.^74^ Clinically, the higher α_2_/α_1_ selectivity of medetomidine results in increased sedation and analgesia compared to xylazine and clonidine due to the lesser extent of α_1_-AR activation.^10^ In addition to its high selectivity among ARs, medetomidine also exhibits high specificity for α_2_-ARs over other key targets, with negligible affinity for β-adrenoreceptors, serotonin, muscarinic, dopamine, or tryptamine receptors in receptor binding experiments and organ tissue baths.^26^ It is also worth mentioning that medetomidine has been shown to bind to the I1-imidazoline receptor, however the pharmacological significance of this binding is not well validated.^75−78^

**Table 2:** α_2_-AR Agonist Selectivity Profiles^79^

	*K*_*i*_ (nM)		
compound	α_1_	α_2_	selectivity ratio (α_2_/α_1_)
medetomidine	1750 ± 567	1.08 ± 0.23	1620
xylazine	30300 ± 1720	194 ± 35.3	160
clonidine	713 ± 109	3.20 ± 1.18	220

### Crystal Structures

**Figure 3:**
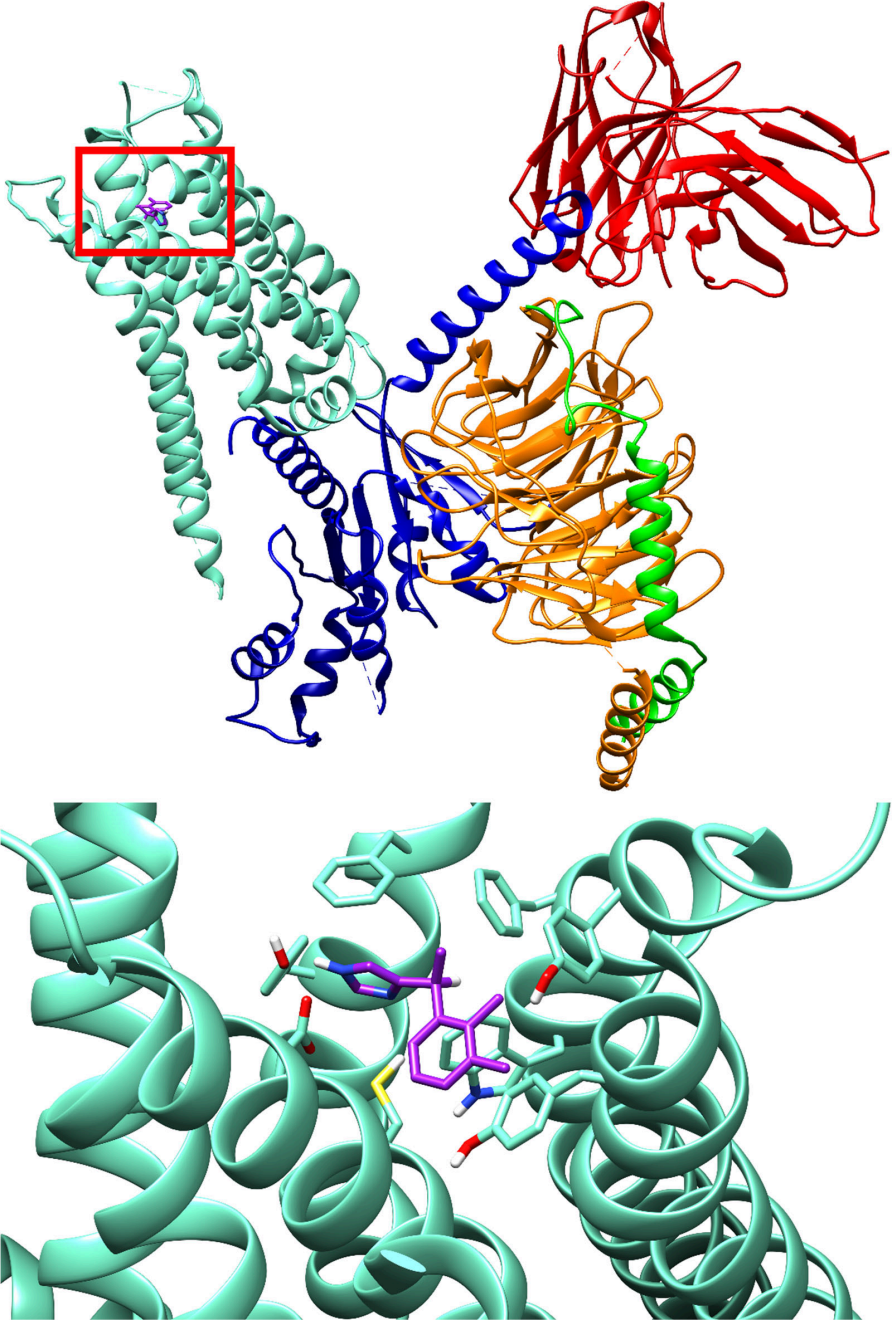
Crystal structure of dexmedetomidine bound to the α_2A_-adrenergic receptor signaling complex (PDB ID: 7EJA).^81^ Created using Chimera.^82^

To date, two cryo-EM structures of dexmedetomidine complexed to the α_2B_-AR (PDB ID: 6K41 and 6K42)^80^ and one crystal structure of dexmedetomidine complexed to the α_2A_-AR (PDB ID: 7EJA, Figure 3)^81^ have been solved. Dexmedetomidine binds to the orthosteric site of these adrenoceptors, and cocrystallization of other α_2A_-AR agonists, such as norepinephrine, have demonstrated that these compounds adopt similar conformations.^81^ The binding of dexmedetomidine to α_2A_ and α_2B_ is primarily facilitated through π–π and hydrophobic interactions with adjacent phenylalanine and tyrosine residues, as well as a key hydrogen bond with a nearby aspartate residue (ASP128 and ASP291 for α_2A_ and α_2B_, respectively).^80,81^

## Clinical Uses

While medetomidine is primarily utilized in veterinary medicine, there is limited research on its effects in humans. A 1989 study found that intravenous doses up to 120 μg were well tolerated in healthy male volunteers, with no reported adverse effects.^83^ Key findings from the study included a dose-dependent reduction of norepinephrine production by up to 75%, as well as sedative effects observed at higher doses (100–120 μg), with onset within 15–45 min and lasting up to 4 h.^83^ Additionally, administration of medetomidine resulted in decreased blood pressure and a reduction in heart rate. These effects are similar to clonidine and xylazine.^83^

Clinical trials have demonstrated that dexmedetomidine significantly reduces the need for rescue sedation and analgesia in adults requiring postsurgical mechanical ventilation and sedation when compared to benzodiazepines.^84^ Dexmedetomidine is also a preferred agent within the pediatric intensive care unit (PICU) setting for the management of sedation, agitation, pain, and delirium.^85^ It has also been clinically used to manage emergence delirium following anesthesia in both adult and pediatric patients, owing to its anxiolytic and sedative properties.^20^

## Current Issues/Concerns

**Figure 4:**
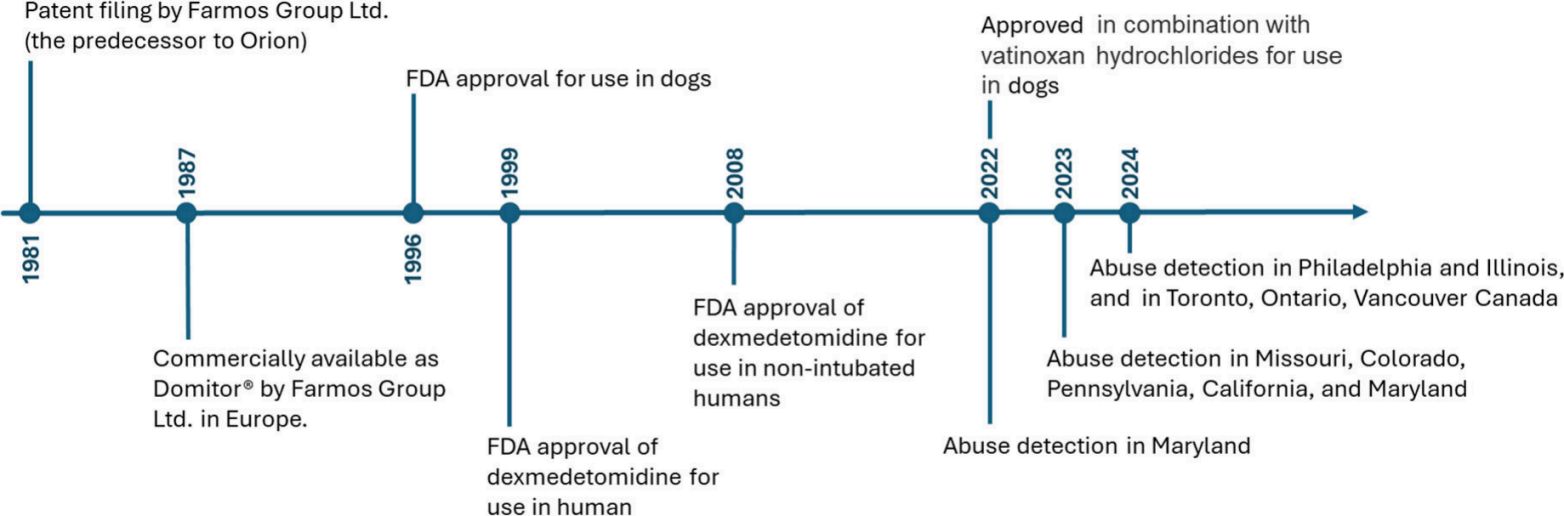
Timeline of the development and recreational use of medetomidine.

Since July 2022, medetomidine has been detected in several seized drug samples across the state of Maryland, as well as in drug paraphernalia and illicit drug seizures submitted to public health and law enforcement agencies.^22,27^ This was a result of a new public health-public safety partnership program initiated in the state of Maryland in October 2021. This partnership enabled the rapid detection and identification of drugs of misuse across the state on several occasions in late 2022, including the detection of medetomidine as an adulterant alongside fentanyl and xylazine.^22^ In midto-late 2023, medetomidine started to appear in toxicology specimens of patients admitted to emergency departments after suspected opioid overdoses in Missouri, Colorado, Pennsylvania, California, and Maryland.^23^ Subsequently, in early 2024, a spike in medetomidine in the recreational drug supply of Canada was detected in drug samples and toxicology specimens originating from Toronto, Ontario and Vancouver, British Columbia. In mid-2024, medetomidine was identified in illicit drug samples in Philadelphia and Illinois, causing “large-scale overdose” and adverse events. On these occasions, medetomidine was detected alongside fentanyl and xylazine, as well as in combination with heroin, fentanyl analogs, and cocaine.^28^ With medetomidine detected in recreational drug samples or patient toxicology specimens across 12 US states and 2 Canadian provinces at the time of this writing, government officials and medical professionals are concerned about the continued spread of this drug across North America (Figure 4).^86^

The emergence of α_2_-AR agonists such as medetomidine and xylazine in illicit drug mixtures presents a complex challenge to public health efforts addressing the opioid crisis. The synergistic interaction between α_2_-AR agonists and opioids not only intensifies the desired effects for users but also significantly increases the risk of severe adverse outcomes, including potentially fatal overdoses.^24^ Furthermore, the addition of these veterinary tranquilizers complicates overdose treatment protocols, as standard opioid antagonists like naloxone may not fully reverse the effects of these drug combinations.^27,87^ It remains unclear whether medetomidine has the potential to exacerbate the skin and soft tissue damage that is associated with xylazine/fentanyl mixtures, however, clinical use of dexmedetomidine (either intramuscularly or intravascularly) has not been linked to wound formation.^22^ It is worth mentioning skepticism has been raised concerning the root cause of skin necrosis seen with xylazine/fentanyl mixtures, particularly whether it results from the involvement of xylazine or from unsterile injection practices.^88^ A recent report documenting the clinical effects of medetomidine in combination with fentanyl and xylazine evaluated in emergency departments reported a range of symptoms including hypotension, acidosis, and hemodynamic complications.^89^ While this report is the first informative report of clinical presentations of this dangerous drug mixture, it is hard to definitively conclude the exact cause of each of these symptoms and thus requires further examination. As the landscape of illicit drug use continues to evolve, understanding the pharmacological interactions and developing targeted interventions for these polydrug combinations becomes increasingly critical in mitigating the ongoing opioid crisis.

## Conclusion

The adulteration of illicit drugs, namely potent narcotics such as fentanyl, with CNS depressants (e.g., medetomidine and xylazine), continues to be a major public health concern. While the procedure for reversing opioid overdose is well-established (i.e., administration of naloxone), there are currently no FDA approved treatments to reverse the α_2_-AR depressant effects of medetomidine and other α_2_-AR agonists.

The rationale for the emergence of medetomidine in the illicit drug supply is currently unclear. Current speculation has attributed this to the ability of medetomidine to potentiate opioid effects, thereby requiring less fentanyl to achieve a similar high and circumventing the restrictive bans on the unlawful import of xylazine and its synthetic precursors that were put in place by the FDA in 2023 since the entrance of xylazine in the illegal drug trade.^90^ The source of medetomidine for illicit use is also uncertain at this time, with possibilities including interception of veterinary supplies or synthesis in clandestine laboratories. The lack of details surrounding this emerging trend renders it difficult to address this new facet of the ever-evolving opioid crisis. Successfully combating this emerging public health threat will require a collaborative and multipronged approach, likely including prevention education, harm reduction, the design and implementation of standardized medetomidine detection methods to better provide suitable patient treatment plans, and the development of suitable treatments to reverse the effects of α_2_-AR agonist overdose.
